# Real-world costs of autosomal dominant polycystic kidney disease in the Nordics

**DOI:** 10.1186/s12913-017-2513-8

**Published:** 2017-08-15

**Authors:** Daniel Eriksson, Linda Karlsson, Oskar Eklund, Hans Dieperink, Eero Honkanen, Jan Melin, Kristian Selvig, Johan Lundberg

**Affiliations:** 1 Quantify Research, Hantverkargatan 8, 112 21 Stockholm, Sweden; 2 Odense University Hospital, Department of Nephrology, Sdr. Boulevard 29, DK-5000 Odense C, Denmark; 3 Helsinki University Central Hospital, Department of Medicine, Division of Nephrology, Haartmaninkatu 4, P.O Box 372, FIN-00029 HUS Helsinki, Finland; 4 Uppsala University Hospital, Department of Nephrology, 751 85 Uppsala, Sweden; 5 Vestre Viken Hospital Trust, Department of Nephrology, Postboks 800 3004, Drammen, Norway; 6 Otsuka Pharma Scandinavia, Birger Jarlsgatan 27, 111 45 Stockholm, Sweden

**Keywords:** Polycystic kidney diseases, ADPKD, Health Care Costs, Health Expenditures, Cost of Illness

## Abstract

**Background:**

There is limited real-world data on the economic burden of patients with autosomal dominant polycystic kidney disease (ADPKD). The objective of this study was to estimate the annual direct and indirect costs of patients with ADPKD by severity of the disease: chronic kidney disease (CKD) stages 1–3; CKD stages 4–5; transplant recipients; and maintenance dialysis patients.

**Methods:**

A retrospective study of ADPKD patients was undertaken April–December 2014 in Denmark, Finland, Norway and Sweden. Data on medical resource utilisation were extracted from medical charts and patients were asked to complete a self-administered questionnaire.

**Results:**

A total of 266 patients were contacted, 243 (91%) of whom provided consent to participate in the study. Results showed that the economic burden of ADPKD was substantial at all levels of the disease. Lost wages due to reduced productivity were large in absolute terms across all disease strata. Mean total annual costs were highest in dialysis patients, driven by maintenance dialysis care, while the use of immunosuppressants was the main cost component for transplant care. Costs were twice as high in patients with CKD stages 4–5 compared to CKD stages 1–3.

**Conclusions:**

Costs associated with ADPKD are significant and the progression of the disease is associated with an increased frequency and intensity of medical resource utilisation. Interventions that can slow the progression of the disease have the potential to lead to substantial reductions in costs for the treatment of ADPKD.

**Electronic supplementary material:**

The online version of this article (doi:10.1186/s12913-017-2513-8) contains supplementary material, which is available to authorized users.

## Background

Autosomal dominant polycystic kidney disease (ADPKD) is a dominantly inherited systemic disease characterised by progressive growth of renal cysts. Recent studies in Europe estimate the prevalence at around one in 3000 people [1, 2], equivalent to fewer than 200,000 cases in the European Union. While a rare disease overall, ADPKD is one of the most common hereditary diseases.

Clinical symptoms of renal disease can occur at any age but typically begin in the third or fourth decade of life [3]. Kidney volume growth is due to cyst expansion and precedes functional renal deterioration (as measured by glomerular filtration rate [GFR]) by several decades. Compensatory hyperfiltration in surviving nephrons initially maintains renal function near normal values. Around 50% of patients require renal replacement therapy due to kidney failure, which typically develops in the fourth to sixth decade of life [3]. Conventional treatments are tailored to reduce morbidity due to complications of the disease [4]. However, new treatment options slowing down the progression of the disease have now become available [5]. Transplantation is the treatment of choice for end-stage renal disease (ESRD) in ADPKD [4]. Still only a limited number of patients with ESRD undergo transplantation instead of dialysis as initial renal replacement therapy [6].

There is sparse information on the economic burden of ADPKD. One study showed an association between direct medical costs and advanced renal dysfunction in patients with polycystic kidney disease who were free of indications of dialysis or transplantation at baseline [7]. A recent study of early-stage ADPKD patients with normal kidney function, found that these patients added a sizable economic burden to the health care system relative to the general population [8]. In a cross-sectional analysis, ADPKD patients, compared to chronic kidney disease (CKD) patients, were found to be younger and generally healthier [9]. However, kidney-related complications and major kidney procedures were more common among ADPKD patients. Further, a retrospective study of medical resource utilisation in ESRD showed that ADPKD patients were younger at dialysis initiation and had lower medical costs compared to control patients with ESRD etiologies other than ADPKD [10].

Cost estimates of ADPKD have been predominantly based on US reimbursement claims data and focused on direct medical resource utilisation for a subset of the population. The objective of this study was to estimate the annual direct and indirect costs of patients with ADPKD, by severity of the disease, in the Nordics.

## Methods

### Study design

This was a cross-sectional study of patients with ADPKD based on data collected from medical charts and patient self-administered questionnaires. Nine nephrology clinics participated; four in Denmark, one in Finland, two in Norway and two in Sweden. Between April and December 2014, we screened and enrolled convenience samples of subjects from each clinic. Patients were recruited by phone or in-person during routine clinical care.

Ethics approvals for the study were granted by the Helsinki University Hospital Ethical Review Board, the Regional Committee of Medical and Health Research Ethics in Oslo (REC South East) and the Regional Ethical Review Board in Stockholm. While the study was reported to the Danish Health and Medicines Authority, formal ethics approval was not required due to the non-interventional design. The study protocol and consent procedures were also reviewed and approved by the participating clinics.

### Sample and inclusion criteria

Subjects were enrolled into four mutually exclusive strata using a hierarchical approach:

1. maintenance dialysis: patients currently on dialysis with or without transplanted kidney
2. transplant recipients: patients with a functioning transplanted kidney, currently not on dialysis
3. CKD stages 4–5: patients not currently on dialysis/no previous transplant
4. CKD stages 1–3: patients not currently on dialysis/no previous transplant

Disease severity among ADPKD patients was determined using the estimated GFR (eGFR), as calculated by each respective laboratory; eGFR <30 ml/min/1.73m^2^ for CKD stages 4–5 and eGFR ≥30 ml/min/1.73m^2^ for CKD stages 1–3. The most recent laboratory value was used to establish disease severity at enrolment date. Enrolment of patients was tracked in order to achieve a balanced recruitment across the four groups.

Subjects were eligible for enrolment in the study if they were 18 years of age or older and had been managed for ADPKD at the clinic during the past 12 months. Furthermore, participants were required to have had an eGFR value recorded in the past 12 months (not applicable if on dialysis). Subjects were excluded if they had been involved in a clinical trial in the past 12 months that resulted in a change in the standard of care received. Patients on maintenance dialysis were required to have had initiated dialysis at least six months prior to enrolment. Similarly, patients with a working kidney transplant were required to have had undergone the transplant procedure at least six months prior to enrolment. Finally, informed written consent was required for participation in the study.

### Data collection

Data were extracted from medical charts using a standardised case report form (CRF) and complemented with a self-administered questionnaire [see Additional files 1 and 2]. The CRF and questionnaire were matched for each subject using anonymised subject identifiers.

The standardised CRF allowed for uniform collection of demographic data, disease history and annual ADPKD-related resource utilisation. The CRF covered the 12-month period prior to patient enrolment (enrolment date).

The questionnaire, completed by patients, included complementary questions on ADPKD-related healthcare services received in the past four weeks outside of the primary nephrology clinic, including informal care. Indirect morbidity measures in terms of time missed from work and impairment of work productivity were obtained using the Work Productivity and Activity Impairment (WPAI:GH) questionnaire [11].

### Cost estimation

A societal perspective was used to estimate total costs. We summarised both direct and indirect annual costs related to ADPKD. Resources used in the past 12 months were quantified for each patient and multiplied by unit costs to derive total annual costs. Unit costs for healthcare services (e.g. primary care visit or blood transfusion) were obtained from local and national pricelists as presented in Table 1. Additional costs were derived from public reports and national statistics offices (e.g. daily cost of peritoneal dialysis or gross earnings/employment rates).

**Table 1:** Data sources for estimating costs

Type of data	Country	Source
Direct costs^a^	Denmark	Danish Medicines Agency [13] Statens Serum Institut [14] The Capital Region of Denmark [15] Rigshospitalet [16]
	Finland	Pharmaceuticals Pricing Board [17] The Social Insurance Institution of Finland [18] The Hospital District of Helsinki and Uusimaa [19, 20] National institute for health and welfare [21] Kuopio University Hospital [22]
	Norway	Norwegian Medicines Agency [23] Norwegian Directorate of Health [24, 25] Ministry of Health and Care Services [26]
	Sweden	Dental and Pharmaceutical Benefits Agency [27] Swedish Association of the Pharmaceutical Industry [12] Region Skåne [28] Stockholm County Council [29]
Indirect costs	Denmark	Statistics Denmark [30, 31] Eurostat [32] KPMG [33]
	Finland	Statistics Finland [34, 35]
	Norway	Statistics Norway [36–38] KPMG [33]
	Sweden	Statistics Sweden [39, 40] Swedish Tax Agency [41]

^a^Transportation costs (to and from haemodialysis) were based on answers in the self-administered questionnaire: taxi, 15 km; public transport, 30 min duration; car, 30 km

Medical resource utilisation was analysed in terms of hospitalisation, outpatient visits, primary care visits, transportation, surgical procedures, diagnostic tests and pharmacotherapy. Pharmacotherapy costs were estimated using conservative dosage estimates as per the drug label [12] for the following classes: antihypertensives, phosphate binders, erythropoiesis-stimulating agents (ESAs), analgesics for kidney pain, vitamin D analogues and immunosuppressive agents.

Indirect costs included informal care and productivity loss. Cost of informal care was based on hours of help from family and friends in the patient’s home and calculated using data on average national gross earnings. Productivity loss was estimated using the human capital approach, taking the patient’s perspective and counting every lost hour of work as lost production and income [42]. Age- and sex-dependent gross earnings and employment rates were obtained from official statistics offices in each country, with employment overheads and benefits added on top. It was assumed that ADPKD patients would have had the same employment rate as the general population had they not been ill.

Annual cost estimates were derived using national cost data and expressed in the local currency of each respective country (2014 values).

### Statistical analyses

Summary statistics were calculated, including means and standard deviations (SDs) for continuous variables and frequency distributions for categorical variables. We presented costs as means and used non-parametric bootstrapping procedures to derive 95% confidence intervals. Differences across strata were evaluated using the Kruskal–Wallis and χ^2^/Fisher’s exact tests as appropriate. Resource utilisation in the past four weeks, as captured in the self-administered questionnaire, was extrapolated to one year. Data management and analysis were performed using Stata 12.1 (StataCorp LP, College Station, TX, USA).

## Results

### Demographic and clinical characteristics

A total of 266 patients were contacted. Of these 243 (91%) provided consent to participate and were enrolled into the four disease strata: CKD stages 1–3 (n = 64), CKD stages 4–5 (n = 55), transplant (n = 61), and dialysis (n = 63). Overall, 241 (99%) of participants completed the questionnaire.

Dialysis and transplant patients tended to be older than patients in earlier stages of the disease; those younger than 65 years were 80% in patients with CKD stages 1–3, 76% in CKD stages 4–5, 54% in dialysis patients and 70% in transplant recipients. Mean age for initiation of dialysis was 59 years in the dialysis stratum and the average age at the time of kidney transplantation was 52 years. Among those on dialysis, only two patients (3%) had received both haemodialysis and peritoneal dialysis in the past 12 months. No differences between disease strata were seen in sex and BMI (Table 2). Employment rates were lowest in the dialysis stratum (21%), with corresponding rates of 44% in transplant recipients, 49% in CKD stages 4–5 and 63% in CKD stages 1–3.

**Table 2:** Patient characteristics at enrolment date

Patient characteristic	CKD 1–3 (n = 64)	CKD 4–5 (n = 55)	Dialysis (n = 61)	Transplant (n = 63)	*P* value
Country, n (%)^a^					<0.0001
Denmark	26 (41)	32 (58)	32 (52)	28 (44)	
Sweden	19 (30)	12 (22)	14 (23)	13 (21)	
Norway	19 (30)	11 (20)	4 (7)	16 (25)	
Finland	0 (0)	0 (0)	11 (18)	6 (10)	
Sex (female), n (%)	38 (59)	29 (53)	33 (54)	31 (49)	0.7144
Age (years), mean ± SD^b^	52 ± 13	57 ± 12	64 ± 10	59 ± 10	<0.0001
BMI (≥30 kg/m^2^), n (%)	10 (16)	11 (20)	15 (25)	14 (22)	0.7667
Currently employed, n (%)	40 (63)	27 (49)	13 (21)	28 (44)	<0.0001
Currently employed (aged <65 years), n (%)	40 (78)	27 (64)	12 (38)	26 (59)	<0.0001
Comorbidities (≥1), n (%)	43 (67)	44 (80)	61 (100)	45 (76)	<0.0001
Dialysis in the past 12 months, n (%) ^a^	.	.	61 (100)	5 (8)	<0.0001
Haemodialysis	.	.	51 (84)	5 (100)	1.0000
Peritoneal dialysis	.	.	12 (20)	0 (0)	0.5754

*P* values calculated with χ^2^ test unless otherwise specified

*SD* standard deviation, *BMI* body mass index

^a^Fisher’s exact test

^b^Kruskal–Wallis test

### Medical resource utilisation

Medical resource utilisation differed substantially between disease strata (Table 3). In general, dialysis patients had the highest number of hospitalisations and outpatient visits, followed by transplant recipients and other dialysis-independent patients. This difference, however, was not observed for primary care visits, as reported in the self-administered questionnaire.

**Table 3:** Annual resource utilisation

Mean resource utilisation, past 12 months ± SD	CKD 1–3 (n = 64)	CKD 4–5 (n = 55)	Dialysis (n = 61)	Transplant (n = 63)	*P* value
Number of hospitalisations	0.2 ± 0.6	0.5 ± 1.1	1.8 ± 2.3	0.6 ± 1.0	<0.0001
Number of hospital days	0.9 ± 3.1	2.3 ± 6.9	9.2 ± 13.6	4.4 ± 10.2	<0.0001
Number of hospital days (at least one hospitalisation)	6.9 ± 6.5	8.7 ± 11.6	15.7 ± 14.7	12.4 ± 14.1	0.1878
Number of outpatient visits^a^	5.2 ± 10.5	8.2 ± 17.1	15.2 ± 24.1	11.6 ± 13.7	<0.0001
Number of primary care visits^b^	2.2 ± 5.5	3.8 ± 16.8	1.9 ± 8.0	1.2 ± 3.8	0.6401
Number of surgical procedures	0.1 ± 0.4	0.3 ± 0.7	1.6 ± 3.1	0.6 ± 1.3	<0.0001
Hours of help: Healthcare professional^b^	27.0 ± 149.8	1.8 ± 9.6	17.1 ± 61.2	6.4 ± 40.4	0.1155
Hours of help: Home care assistant^b^	0.0 ± 0.0	0.2 ± 1.8	27.6 ± 132.6	0.8 ± 3.8	<0.0001
Hours of help: Family member or friend^b^	3.1 ± 18.2	27.0 ± 84.6	104.8 ± 325.1	11.0 ± 31.0	<0.0001

*P* values calculated with Kruskal–Wallis test

^a^Excluding visits for maintenance dialysis

^b^Based on the past 4 weeks, self-reported

Only 8% of CKD stages 1–3 patients had a surgery related to ADPKD in the past year, compared to 18% of CKD stages 4–5 patients, 29% of transplant recipients and 49% of dialysis patients. Consequently, there was a significant difference in the mean number of surgical procedures in the past year between the disease strata, ranging from 0.1 in patients with CKD stages 1–3 to 1.6 in dialysis patients. Among transplant recipients, 10% had received the transplant in the past year. Similarly, 25% of dialysis patients had initiated treatment in the past year.

Dialysis patients were generally prescribed more drugs compared to the other disease states; 95% of dialysis patients used phosphate binders, 80% used erythropoiesis-stimulating agents (ESAs) and 97% were prescribed vitamin D analogues (Table 4). Analgesics for kidney pain were, however, most common in CKD stages 4–5, used by 27% compared to 16–23% in the other disease strata. Almost all patients with CKD stages 4–5 (98%) were prescribed antihypertensives.

**Table 4:** Annual drug utilisation

Proportion (%) of patients using drug class, past 12 months	CKD 1–3 (n = 64)	CKD 4–5 (n = 55)	Dialysis (n = 61)	Transplant (n = 63)	*P* value
Antihypertensives	84	98	84	87	0.0275
Phosphate binders	0	21	95	14	<0.0001
ESAs	2	13	80	15	<0.0001
Analgesics for kidney pain^a^	17	28	25	17	0.4149
Vitamin D analogs^a^	14	57	97	43	<0.0001
Immunosupressants^a^	0	0	7	100	<0.0001
Other drugs	19	36	90	41	<0.0001

*P* values calculated with Fisher’s exact test unless otherwise specified

*ESA* Erythropoiesis-stimulating agent

^a^χ^2^ test

Among dialysis patients 59% travelled by taxi to receive their treatment, while 35% drove and 6% used public transport. Forty-three percent travelled for at least 30 min one-way to receive treatment.

### Activity and work impairment

The levels of general daily activity impairment and productivity impairment due to health problems differed with disease severity. Activity impairment was highest among dialysis patients with 53% but also substantial at 30% in both patients with CKD stages 4–5 and among transplant recipients (Table 5). Among those employed, an average of 4–26% of work time was missed due to health problems, while patients estimated 7–26% of time lost while at work, depending on disease severity. Taken together, overall work impairment due to health was significantly different between disease strata. Work impairment was highest among dialysis patients (42%), followed by CKD stages 4–5 (23%), transplant recipients (16%) and CKD stages 1–3 (9%).

**Table 5:** Productivity loss

WPAI-GH^a^, percent (%) ± SD	CKD 1–3 (n = 61)	CKD 4–5 (n = 53)	Dialysis (n = 57)	Transplant (n = 63)	*P* value
Activity impairment due to health	16.7 ± 24.4	29.4 ± 28.0	52.6 ± 27.2	30.4 ± 27.5	<0.0001
Overall work impairment due to health	8.7 ± 14.6	22.8 ± 28.7	41.8 ± 33.5	16.4 ± 23.1	0.0025
Work time missed due to health (absenteeism)	4.2 ± 17.3	8.3 ± 18.9	25.9 ± 32.8	4.6 ± 19.6	0.0014
Impairment while working due to health (presenteeism)	7.4 ± 12.2	18.8 ± 24.1	25.8 ± 23.9	15.0 ± 20.8	0.0109

*P* values calculated with Kruskal–Wallis test

*WPAI-GH* Work Productivity and Activity Impairment-General Health

^a^Patients were asked to estimate impairment in the past 7 days (recall period)

### Annual costs associated with ADPKD

Costs are presented by disease severity and expressed in each respective local currency (Tables 6, 7, 8 and 9). Average total annual costs were highest for dialysis patients, followed by transplant recipients, patients in CKD stages 4–5 and CKD stages 1–3 (P < 0.0001, for all countries). Compared to CKD stages 1–3, annual costs were almost twice as high in CKD stages 4–5, two to three times higher in transplant recipients, and seven to nine times higher in dialysis patients. Differences between disease strata were even more pronounced when looking at direct costs alone (P < 0.0001, for all countries). Direct costs were almost twice as high in patients with CKD stages 4–5 compared to stages 1–3, but around six times higher among transplant recipients and 21 times higher among dialysis patients. Direct medical costs were substantial among dialysis patients, with routine dialysis care alone accounting over half of total costs. Productivity loss was a driver of costs across all stages of ADPKD, and especially substantial at around two-thirds of total costs in patients with CKD stages 1–3 and 4–5.

**Table 6:** Annual costs in Danish krone (Denmark)

Costs in DKK, mean (95% CI)	CKD 1–3 (n = 64)	CKD 4–5 (n = 55)	Dialysis (n = 61)	Transplant (n = 63)	*P* value
Direct costs	28,022 (14,728–50,835)	47,203 (35,863–63,990)	667,362 (623,398–720,640)	196,114 (159,055–237,980)	<0.0001
Hospitalisations	4736 (1611–10,505)	12,224 (4810–24,391)	50,954 (34,240–71,642)	23,881 (12,072–40,151)	<0.0001
Outpatient care visits	5596 (3810–7867)	9483 (6889–14,515)	15,802 (11,175–21,341)	14,219 (10,992–18,453)	<0.0001
Primary care visits	1558 (779–2761)	2719 (712–7252)	1144 (163–2942)	791 (317–1741)	.5094
Surgical procedures	2183 (125–7519)	6938 (2660–13,242)	31,596 (18,141–50,394)	4228 (1812–7559)	<0.0001
Diagnostic tests	1591 (1055–2225)	1803 (1239–2542)	6081 (4851–7544)	3464 (2374–5095)	<0.0001
Home care/medical assistance	9838 (38–28,949)	717 (62–1940)	12,399 (4417–23,648)	2503 (313–7919)	.0001
Routine dialysis care	–	–	441,221 (417,652–462,446)	14,377 (3783–28,905)	<0.0001
Haemodialysis transportation	–	–	41,146 (33,117–49,306)	214 (0–1068)	<0.0001
Drug use	2520 (1404–3856)	13,318 (9605–17,716)	67,020 (57,869–79,028)	132,438 (110,082–158,657)	<0.0001
Antihypertensives	391 (295–507)	476 (394–559)	343 (272–420)	401 (303–534)	.0560
Phosphate binders	–	1351 (656–2160)	10,551 (8212–13,158)	521 (123–1242)	<0.0001
ESAs	60 (0–245)	3366 (1286–5953)	23,281 (19,497–26,558)	3449 (1459–6102)	<0.0001
Analgesics for kidney pain	17 (5–39)	90 (21–253)	182 (26–534)	13 (2–37)	.1069
Vitamin D analogues	2004 (937–3379)	6955 (5201–8861)	13,309 (12,162–14,207)	4593 (3129–6257)	<0.0001
Immunosupressants	–	–	5699 (312–14,111)	122,984 (100,943–149,412)	<0.0001
Other drugs	48 (11–110)	1081 (157–2500)	13,655 (10,825–16,513)	477 (194–932)	<0.0001
Indirect costs	51,523 (32,278–75,631)	94,631 (65,117–126,721)	100,970 (67,789–132,323)	81,688 (55,334–110,676)	.0726
Productivity loss	51,224 (31,835–75,332)	92,083 (63,079–123,547)	91,373 (59,420–122,164)	80,647 (54,460–109,503)	.3032
Informal care	299 (0–896)	2548 (873–5141)	9597 (3801–19,465)	1041 (415–1891)	<0.0001
Total costs	79,544 (54,826–109,204)	141,834 (105,601–181,449)	768,332 (707,301–830,831)	277,802 (227,251–333,023)	<0.0001

*P* values calculated with Kruskal–Wallis test

*DKK* Danish krone, *ESA* erythropoiesis-stimulating agent, *CI* confidence interval (bias corrected)

**Table 7:** Annual costs in euro (Finland)

Costs in EUR, mean (95% CI)	CKD 1–3 (n = 64)	CKD 4–5 (n = 55)	Dialysis (n = 61)	Transplant (n = 63)	*P* value
Direct costs	3676 (2223–6190)	5883 (4588–7701)	64,811 (60,460–70,417)	20,305 (16,228–25,166)	<0.0001
Hospitalisations	507 (172–1125)	1309 (515–2611)	5455 (3666–7670)	2557 (1283–4299)	<0.0001
Outpatient care visits	1159 (843–1569)	2057 (1606–2871)	3203 (2329–4230)	3197 (2502–4155)	<0.0001
Primary care visits	237 (117–403)	414 (84–1097)	174 (50–548)	121 (48–265)	.5094
Surgical procedures	249 (15–794)	511 (154–1098)	2562 (1211–5119)	1009 (435–1956)	<0.0001
Diagnostic tests	191 (125–263)	237(166–321)	659 (511–843)	354 (251–496)	<0.0001
Home care/medical assistance	1048 (4–3049)	74 (5–204)	1093 (384–2044)	260 (28–837)	.0002
Routine dialysis care	–	–	42,900 (40,609–44,964)	1398 (368–2810)	<0.0001
Haemodialysis transportation	–	–	4090 (3184–5080)	13 (0–25)	<0.0001
Drug use	284 (175–419)	1281 (1008–1600)	4675 (4040–5512)	11,396 (9404–13,944)	<0.0001
Antihypertensives	68 (54–86)	113 (95–132)	73 (60–86)	96 (78–117)	.0020
Phosphate binders	–	225 (103–362)	1266 (1006–1554)	65 (15–148)	<0.0001
ESAs	4 (0–15)	204 (78–361)	1413 (1181–1611)	209 (89–370)	<0.0001
Analgesics for kidney pain	3 (1–7)	14 (5–30)	12 (4–25)	4 (0–12)	.1329
Vitamin D analogues	198 (93–335)	689 (516–878)	1318 (1207–1407)	455 (310–620)	<0.0001
Immunosupressants	–	–	440 (26–1076)	10,469 (8516–12,955)	<0.0001
Other drugs	10 (3–27)	36 (17–61)	154 (126–183)	99 (48–171)	<0.0001
Indirect costs	4863 (2986–7132)	9904 (6738–13,319)	7674 (5195–10,042)	7585 (5125–10,494)	.0925
Productivity loss	4835 (2959–7104)	9667 (6586–13,018)	6783 (4586–8815)	7488 (5058–10,382)	.2742
Informal care	28 (0–83)	237 (79–475)	891 (353–1807)	97 (38–176)	<0.0001
Total costs	8539 (6042–11,631)	15,787 (12,006–20,008)	72,486 (67,053–79,025)	27,890 (22,669–33,722)	<0.0001

*P* values calculated with Kruskal–Wallis test

*EUR* euro, *ESA* erythropoiesis-stimulating agent, *CI* confidence interval (bias corrected)

**Table 8:** Annual costs in Norwegian krone (Norway)

Costs in NOK, mean (95% CI)	CKD 1–3 (n = 64)	CKD 4–5 (n = 55)	Dialysis (n = 61)	Transplant (n = 63)	*P* value
Direct costs	38,676 (18,712–69,343)	80,145 (51,159–118,538)	851,277 (765,334–959,286)	185,108 (131,915–251,557)	<0.0001
Hospitalisations	12,898 (4387–28,610)	33,291 (13,098–66,704)	138,766 (93,249–195,108)	65,036 (32,637–109,347)	<0.0001
Outpatient care visits	4425 (3355–5859)	8085 (6588–10,612)	12,050 (8956–15,673)	12,840 (10,094–16,706)	<0.0001
Primary care visits	691 (345–1224)	1205 (246–3192)	507 (145–1594)	351 (140–772)	.5094
Surgical procedures	4209 (89–15,018)	23,660 (10,359–40,439)	106,888 (67,415–153,360)	6798 (1790–14,265)	<0.0001
Diagnostic tests	1652 (1100–2273)	1959 (1417–2622)	6423 (5132–8002)	3359 (2450–4587)	<0.0001
Home care/medical assistance	12,317 (48–36,245)	885 (66–2396)	14,151 (5074–27,170)	3090 (359–9872)	.0002
Routine dialysis care	–	–	495,052 (468,607–518,867)	16,131 (4245–32,431)	<0.0001
Haemodialysis transportation	–	–	32,460 (25,631–39,573)	128 (0–256)	<0.0001
Drug use	2483 (1472–3707)	11,060 (8445–14,068)	44,980 (39,925–51,304)	77,375 (64,093–95,936)	<0.0001
Antihypertensives	530 (435–640)	715 (621–808)	522 (433–617)	567 (461–687)	.02459
Phosphate binders	–	1158 (565–1850)	8072 (6345–9932)	418 (102–944)	<0.0001
ESAs	36 (0–148)	2036 (778–3600)	14,079 (11,772–16,055)	2086 (827–3610)	<0.0001
Analgesics for kidney pain	16 (6–31)	103 (33–251)	104 (29–256)	33 (3–98)	.1053
Vitamin D analogues	1833 (857–3090)	6360 (4761–8104)	12,170 (11,121–12,991)	4200 (2861–5722)	<0.0001
Immunosupressants	–	–	2741 (290–6569)	69,324 (56,582–87,711)	<0.0001
Other drugs	68 (19–164)	688 (181–1422)	7292 (5804–8788)	746 (353–1326)	<0.0001
Indirect costs	111,441 (70,268–157,539)	204,324 (143,043–268,451)	215,588 (144,283–280,047)	182,164 (125,099–242,812)	.0604
Productivity loss	110,892 (70,085–157,480)	199,644 (139,793–263,028)	197,961 (129,783–259,106)	180,251 (123,425–241,038)	.2452
Informal care	548 (0–1645)	4680 (1603–9442)	17,627 (7001–35,752)	1913 (761–3473)	<0.0001
Total costs	150,117 (104,759–202,958)	284,469 (206,680–373,107)	1,066,865 (950,458–1,204,094)	367,272 (278,949–466,269)	<0.0001

*P* values calculated with Kruskal–Wallis test

*NOK* Norwegian krone, *ESA* erythropoiesis-stimulating agent, *CI* confidence interval (bias corrected)

**Table 9:** Annual costs in Swedish krona (Sweden)

Costs in SEK, mean (95% CI)	CKD 1–3 (n = 64)	CKD 4–5 (n = 55)	Dialysis (n = 61)	Transplant (n = 63)	*P* value
Direct costs	28,820 (16,123–50,689)	48,624 (36,718–65,151)	712,482 (668,060–766,530)	173,199 (135,833–218,165)	<0.0001
Hospitalisations	3812 (1297–8456)	9840 (3871–19,716)	41,015 (27,561–57,668)	19,223 (9717–32,319)	<0.0001
Outpatient care visits	5878 (4291–7943)	10,457 (8193–14,512)	16,221 (11871–21,402)	16,288 (12,728–21,120)	<0.0001
Primary care visits	1178 (589–2088)	2056 (539–5484)	865 (124–2225)	598 (120–1077)	.5094
Surgical procedures	3349 (187–10,713)	7135 (1620–15,769)	31,431 (16,065–51,975)	8131 (3603–14,557)	<0.0001
Diagnostic tests	2847 (2244–3509)	5810 (4535–7332)	25,140 (21,308–29,294)	10,987 (8119–14,700)	<0.0001
Home care/medical assistance	9442 (37–27,461)	675 (47–1850)	10,525 (3734–20,134)	2359 (263–7537)	.0002
Routine dialysis care	–	–	488,009 (461940–511,484)	15,901 (4185–32,472)	<0.0001
Haemodialysis transportation	–	–	37,145 (30,444–43,657)	269 (0–1344)	<0.0001
Drug use	2313 (1333–3490)	12,651 (9110–16,825)	62,131 (54,647–71,169)	99,443 (80,719–125,735)	<0.0001
Antihypertensives	419 (341–513)	666 (556–784)	503 (415–603)	583 (451–756)	.0282
Phosphate binders	–	1244 (598–1996)	9788 (7577–12,262)	472 (108–1151)	<0.0001
ESAs	63 (0–257)	3535 (1350–6252)	24,450 (20,476–27,892)	3622 (1533–6409)	<0.0001
Analgesics for kidney pain	14 (5–26)	102 (28–255)	78 (21–193)	15 (2–37)	.1032
Vitamin D analogues	1776 (824–2952)	6162 (4613–7852)	11,792 (10,796–12,592)	4069 (2756–5544)	<0.0001
Immunosupressants	–	–	3579 (291–8847)	90,205 (72,049–117,127)	<0.0001
Other drugs	41 (10–95)	942 (135–2184)	11,941 (9447–14,436)	477 (202–909)	<0.0001
Indirect costs	64,259 (39,484–92,072)	128,541 (90,007–169,626)	124,957 (85,289–162,184)	112,688 (77,160–150,663)	.0438
Productivity loss	63,963 (39,446–91,997)	126,019 (88,140–165,959)	115,458 (77,012–150,462)	111,658 (75,795–149,420)	.1842
Informal care	296 (0–887)	2522 (864–5088)	9499 (3762–19,266)	1031 (410–1871)	<0.0001
Total costs	93,079 (64,756–125,857)	177,165 (131,147–227,131)	837,438 (771,457–903,231)	285,887 (228,017–352,229)	<0.0001

*P* values calculated with Kruskal–Wallis test

*SEK* Swedish krona, *ESA* erythropoiesis-stimulating agent, *CI* confidence interval (bias corrected)

## Discussion

In this study we enrolled 243 ADPKD patients from nine nephrology clinics in Denmark, Finland, Norway and Sweden. For these patients we collected and analysed data from medical charts and self-administered questionnaires. Our findings showed that the economic burden of ADPKD was substantial at all levels of disease and that progression of ADPKD was associated with an increased frequency and intensity of medical resource utilisation.

Mean total direct and indirect costs were approximately twice as high in patients with CKD stages 4–5 compared to CKD stages 1–3. Resource utilisation increased substantially as patients progressed to ESRD, with costs among dialysis patients greatly exceeding that of kidney transplant recipients. The use of immunosuppressants accounted for around half of costs in transplant recipients. Similarly, maintenance dialysis care alone accounted for over half of total costs in dialysis patients, who had the highest number of hospitalisations and outpatient visits. Primary care visits were more frequent in earlier stages of the disease. Lost wages due to reduced productivity were large in absolute terms across all disease strata. General daily activity impairment due to health was highest among dialysis patients who reported an average reduction in activity of over 50%. Activity impairment was also substantial in transplant recipients and in patients with CKD stages 4–5, both at around 30%.

Some limitations of our study should be noted. Selection bias may be an issue as with any observational study. No randomisation was performed and primarily patients who actively sought health care were included. Not all patients in earlier stages of the disease are followed by nephrology clinics and the study design limited the inclusion of transplant recipients to those with a functioning transplant, thus potentially underestimating costs in patients with advanced disease. A proportion of patients with ESRD initiated treatment within 12 months of the enrolment date; however, sensitivity analyses revealed an insignificant impact on mean total costs.

Our study adds to the limited and fragmented literature on cost estimates of ADPKD. To our knowledge this is the first study to provide cost data on an ADPKD population that includes both early stages of the disease, stratified by renal function, and patients with ESRD. A further strength of this study is the enrolment of patients with physician-confirmed diagnosis of ADPKD. Furthermore, in addition to data extraction from medical charts, a self-administered questionnaire, including the WPAI:GH, was used to capture resource utilisation outside of the nephrology clinic and to estimate indirect costs in terms of productivity loss and caregiver support. Finally, we achieved a high response rate with 91% of invited patients agreeing to participate in the study.

## Conclusions

We provide a thorough description of the medical resource utilisation and costs associated with ADPKD across all stages of the disease. Our findings confirm the association between economic burden and progression of ADPKD [7]. Costs were highest in dialysis patients, driven by maintenance dialysis care, while the use of immunosuppressants was the main cost component for transplant care. Costs were twice as high in patients with CKD stages 4–5 compared to CKD stages 1–3. Consequently, interventions that can slow the progression of the disease have the potential to lead to substantial reductions in costs for the treatment of ADPKD.

## Additional files

Additional file 1: QR ADPKD CRF 20140331 Final v1.1.pdf – Case report form (CRF) – Questionnaire used to collect data from patients’ medical charts. (PDF 624 kb)

Additional file 2: QR ADPKD Additional Questions 20140205 Eng.pdf – Self-administered questionnaire – Questionnaire administered to patients. (PDF 16 kb)

## Abbreviations

ADPKD: Autosomal dominant polycystic kidney disease
BMI: Body mass index
CKD: Chronic kidney disease
CRF: Case report form
eGFR: Estimated glomerular filtration rate
ESA: Erythropoiesis-stimulating agents
ESRD: End-stage renal disease
SD: Standard deviation
WPAI: Work productivity and activity impairment
