# Supplementary material for: Real-world costs of autosomal dominant polycystic kidney disease in the Nordics
Source: BMC Health Serv Res. 2017 Aug 15;17:560. doi: 10.1186/s12913-017-2513-8 (PMC5556351; doi:10.1186/s12913-017-2513-8)
Supplement: Supplementary file 1 — QR ADPKD CRF 20140331 Final v1.1.pdf – Case report form (CRF) – Questionnaire used to collect data from patients’ medical charts. (PDF 624 kb) [file 12913_2017_2513_MOESM1_ESM.pdf]

Site ID			Subject ID		

Case Report Form (CRF)

Instructions and clarifications appear throughout this form in italic

Instructions

This CRF is used to collect data from medical charts of patients with autosomal dominant polycystic kidney disease (ADPKD). Subjects will be enrolled into the following mutually exclusive strata:

- Dialysis (initiated at least 6 months ago; with or without transplanted kidney)
- Transplant (working transplanted kidney since at least 6 months ago; not on dialysis)
- Chronic kidney disease (CKD) stage 4–5 (not currently on dialysis/no previous transplant)
- CKD stage 1–3 (not currently on dialysis/no previous transplant)

The following eligibility criteria will apply:

- 18 years of age or older
- Managed for ADPKD/CKD at your clinical site during the past 12 months
- Diagnosed with ADPKD and CKD at least 12 months ago
- Estimated Glomerular Filtration Rate (eGFR) laboratory result available in the past 12 months
    - Not applicable if subject is on dialysis
- Has NOT been involved in an investigational drug trial that resulted in a change in standard care received in the past 12 months
    - If there was no change in the care received, the patient is eligible
- If patients is on regular dialysis, date of dialysis initiation was at least 6 months ago
- If patient now has a working kidney transplant, date of transplant was at least 6 months ago
- Receipt of informed consent

Please first review the subject's medical records to complete the eligibility criteria on the following pages. Thereafter, continue filling out the rest of this CRF. The answers to questions 1–6 of the CRF are used to classify subjects into the 4 mutually exclusive strata.

Remember to document enrolled subjects on the *Subject Identification Code List* (assign each subject with a unique site/subject ID number, which is also recorded on each page of this CRF, and use this list to keep track of the number of subjects enrolled into each respective stratum).

Overview of CRF

Apart from *eligibility criteria* and *disease status* on the following pages and the *signature page* on the last page, this CRF can be divided into 3 major parts based on the type of data collected:

I. Data on disease status and current resource use [Strata: *All subjects*]
   - Demographic Data and ADPKD Disease History
   - ADPKD-Related Medical Resource Use (Past 12 Months)
   - ADPKD-Related Medication Use (Past 12 Months)
   - Dialysis-Related Resource Use (Past 12 Months)

II. Data from the period around initiation of dialysis [Strata: *Dialysis*]
   - Dialysis-Related Resource Use (Period around initiation of Dialysis)

III. Data from the period around transplant operation [Strata: *Transplant*]
   - Kidney Transplant-Related Resource Use (Period around the Transplant)

In Part I, data are collected on *all subjects*. Part II only applies to subjects enrolled into the strata *Dialysis*, whereas Part III applies to the *Transplant* strata.

The following figure gives an overview of the data collected and the structure of this CRF.

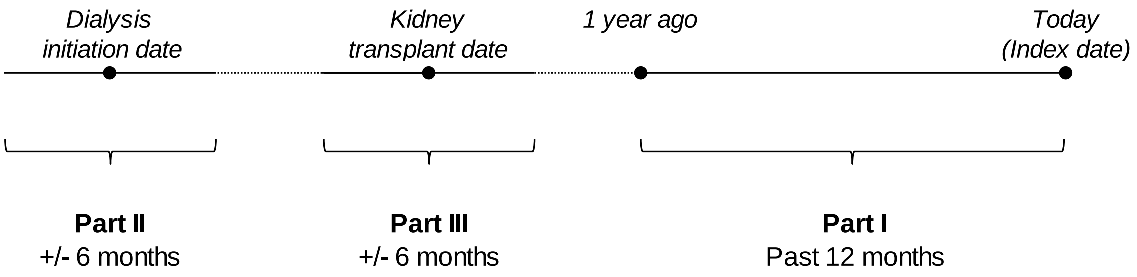

Eligibility Criteria

1. Please complete the following questions and confirm below that the subject meets all inclusion and exclusion criteria

a. Enrolment date (date of data abstraction for the subject)
(End date)

__ __ / __ __ / __ __
D D M M Y Y

b. Date one year prior (subtract 12 months from the date above)
(Start date)

__ __ / __ __ / __ __
D D M M Y Y

c. Please indicate subject's month and year of birth.

If <18 years old, ineligible

__ __ / __ __ __ __
M M Y Y Y Y

d. Has the subject been managed for autosomal dominant polycystic kidney disease (ADPKD)/chronic kidney disease (CKD) at your clinical site during the past 12 months?

☐ Yes
☐ No *(Ineligible)*

e. Has the subject had ADPKD and CKD for at least 12 months?

☐ Yes
☐ No *(Ineligible)*

f. Does the subject have an estimated GFR (eGFR) reported in the past 12 months?

☐ Yes
☐ No *(Ineligible)*
☐ Not applicable (subject is on dialysis)

g. Has the subject been involved in an investigational drug trial in the past 12 months

☐ Yes, that did result in a change in standard of care *(Ineligible)*
☐ Yes, that did not result in a change in standard of care
☐ No

h. If the subject is on regular dialysis, was the date of dialysis initiation at least 6 months ago?

☐ Yes
☐ No *(Ineligible)*
☐ Not applicable (subject is not on dialysis)

i. If the subject now has a working kidney transplant, was the date of transplant procedure at least 6 months ago?

☐ Yes
☐ No *(Ineligible)*
☐ Not applicable (subject does not have a working transplant)

j. Has informed consent been received from the subject?

☐ Yes
☐ No *(Ineligible)*

2. Is the subject eligible for study participation based on the information provided above?

   Please make sure all questions above are answered before confirming eligibility.

   ☐ Yes
   ☐ No

   If yes, please continue filling out the CRF for this subject. The pages that follow are only to be completed for patients that fulfil all eligibility criteria.

ADPKD Disease Status (Classification of Subjects into Strata)

Information collected in this section is used to categorize patients (for enrolment of subjects into strata)

3. Regular dialysis

This refers to regular dialysis due to chronic kidney disease (i.e. not temporary dialysis)

a. Is the subject currently receiving regular dialysis?

☐ Yes
☐ No
☐ Do not know

b. Has the subject previously been receiving regular dialysis?

☐ Yes
☐ No
☐ Do not know

c. If yes to either a) or b) above, please indicate the date(s) when the subject started/ended regular dialysis.

Dialysis start date	End date (if applicable, leave empty if currently on dialysis)
__ __ / __ __ / __ __ __ __<br>D D M M Y Y Y Y	__ __ / __ __ / __ __ __ __<br>D D M M Y Y Y Y
If subject has previously been on dialysis, please provide dates below	
Dialysis start date	End date
__ __ / __ __ / __ __ __ __<br>D D M M Y Y Y Y	__ __ / __ __ / __ __ __ __<br>D D M M Y Y Y Y
__ __ / __ __ / __ __ __ __<br>D D M M Y Y Y Y	__ __ / __ __ / __ __ __ __<br>D D M M Y Y Y Y

If more room is required than the space provided, please use a separate piece of paper.

4. Kidney transplant

a. Has the subject had a kidney transplant?

☐ Yes
☐ No
☐ Do not know

b. If yes, how many kidney transplants have the subject had?

__ __ transplant(s)

c. If yes, please indicate the date(s) of kidney transplant.

Kidney transplant date
__ __ / __ __ / __ __ __ __<br>D D M M Y Y Y Y
If subject has had more than one kidney transplant, please provide dates below
__ __ / __ __ / __ __ __ __<br>D D M M Y Y Y Y
__ __ / __ __ / __ __ __ __<br>D D M M Y Y Y Y

If more room is required than the space provided, please use a separate piece of paper.

5. Renal function assessment (eGFR)

a. Please provide the subject's most recent estimated GFR.

__ __ __ ml/min/1.73 m^2

b. Please indicate the date assessed.

__ __ / __ __ / __ __ __ __
D D M M Y Y Y Y

c. Please indicate the equation/formula used.

☐ MDRD (Modification of Diet in Renal Disease)
☐ CKD-EPI (Chronic Kidney Disease Epidemiology Collaboration)
☐ Cockcroft-Gault
☐ Lund-Malmö
☐ Other, specify: ___________________

6. Based on the information collected above, to which stratum is the subject enrolled?

Each subject is enrolled into one of the four strata in a hierarchical fashion according to the order listed below. Please note that the strata are mutually exclusive.

For example, if a subject is currently on dialysis and also has had a kidney transplant, the subject is enrolled to the stratum "Dialysis" (not to "Transplant").

☐ Dialysis (initiated at least 6 months ago; with or without transplanted kidney)
☐ Transplant (working transplanted kidney since at least 6 months ago; not on dialysis)
☐ CDK stage 4–5 (<30 ml/min/1.73 m^2; not currently on dialysis/no previous transplant)
☐ CKD stage 1–3 (≥30 ml/min/1.73 m^2; not currently on dialysis/no previous transplant)

Part I:
Current Resource Use

Strata: *All subjects*

Demographic Data and ADPKD Disease History

7. Please indicate subject's gender

☐ Male
☐ Female

8. Please indicate subject's height

|_|_|_| cm

9. Please indicate subject's weight

If the patient is on dialysis, please provide the subjects dry weight (the amount of body mass without extra fluid).

|_|_|_| kg

10. Please indicate the subject's most recent blood pressure measurement.

If the patient is on dialysis, please provide blood pressure reading taken <u>after</u> the dialysis session.

Blood pressure (systolic/diastolic)	Date assessed
___ / ___ mm Hg	DD / MM / YYYY

11. Kidney volume/length

a. Has the subject had total kidney volume (TKV) and/or kidney length estimated?

☐ Yes
☐ No
☐ Do not know

b. If yes, please indicate the most recent total kidney volume (TKV) and/or kidney length measurement [provide the largest value(s) if the kidneys differ in TKV and/or length].

Total kidney volume	Date assessed
___ cm^3 or ml	DD / MM / YYYY
**Kidney length**	**Date assessed**
__ . _ cm	DD / MM / YYYY

12. Dialysis/transplantation information

This refers to the patient receiving information about different ways to treat end stage renal disease (ESRD). Please answer this question regardless of whether the subject already is on dialysis/has had a transplant, or not.

a. Has the subject received information about dialysis and transplantation?

- ☐ Yes
- ☐ No
- ☐ Do not know

b. If yes, please indicate the month and year of first receiving information.

		/				
M	M		Y	Y	Y	Y

13. Decision about treatment modality

After the information about treatment options are given, typically a decision is made regarding the modality of treatment. If several decisions have been conducted, please provide information on the most recent decision.

a. Has a decision (e.g. dialysis, transplant, conservative treatment) about how to treat ESRD been made?

- ☐ Yes
- ☐ No
- ☐ Do not know

b. If yes, please indicate the month and year of the decision.

		/				
M	M		Y	Y	Y	Y

c. Has a living kidney donor been accepted for future transplantation?

- ☐ Yes
- ☐ No
- ☐ Do not know

d. If yes, please indicate the month and year of when the donor was identified.

		/				
M	M		Y	Y	Y	Y

14. Preparation for starting haemodialysis

If several operations have been conducted, please provide information on the initial procedure.

a. Has the patient had a vascular access procedure for haemodialysis?

- ☐ Yes
- ☐ No
- ☐ Do not know

b. If yes, please indicate the month and year of the operation.

		/				
M	M		Y	Y	Y	Y

c. If yes, please indicate type(s) of vascular access.

- ☐ Arteriovenous fistula
- ☐ Arteriovenous synthetic graft
- ☐ Central dialysis catheter

15. Preparation for starting peritoneal dialysis

If several operations have been conducted, please provide information on the initial procedure.

a. Has the patient had an access procedure for peritoneal dialysis?

- ☐ Yes
- ☐ No
- ☐ Do not know

b. If yes, please indicate the month and year of the operation.

__ __ / __ __ __ __
M M Y Y Y Y

16. Transplant failure

a. Has the subject had a kidney transplant failure (graft failure, transplant rejection)?

- ☐ Yes (primary non-function, technical failure, acute vascular events, hyper-acute rejection)
- ☐ Yes (acute rejection and/or severe inflammation)
- ☐ Yes (other reason than above)
- ☐ No
- ☐ Do not know

b. If yes, how was the graft failure handled?

Select all that apply.

- ☐ Dialysis treatment started
- ☐ Maintained graft
- ☐ Graft nephrectomy
- ☐ Re-transplantation (planned or performed)

ADPKD-Related Medical Resource Use (Past 12 Months)

17. Does the subject currently have and/or have been diagnosed in the past 12 months with any of the following medical conditions?

(Select all that apply)

☐ Abdominal hernia
☐ Anaemia
☐ Brain aneurysm
☐ Cancer
☐ Colonic diverticulitis
☐ Diabetes mellitus
☐ Haematuria (blood in urine)
☐ Hyperparathyroidism
☐ Hypertension
☐ Nephrolithiasis (kidney stones)
☐ Non-renal cysts
☐ Peritonitis
☐ Proteinuria
☐ Renal haemorrhage
☐ Renal pain
☐ Sepsis
☐ Upper urinary tract infection
☐ Vascular/Cardiac abnormalities
☐ Other diagnosis related to ADPKD or its complications, specify:
____
☐ None of the above

18. Medical surgeries/treatment procedures in the past 12 months

a. In the past 12 months, did the subject have any medical surgeries/treatment procedures?

☐ Yes
☐ No
☐ Do not know

Site ID			Subject ID		

b. If yes, please provide details below.

Surgery/procedure	Did the patient have the procedure in the past 12 months?	Number of procedures over the past 12 months
Acute blood transfusion	☐ Yes<br>☐ No<br>☐ Do not know	▢▢ procedures
Brain aneurysm procedure	☐ Yes<br>☐ No<br>☐ Do not know	▢▢ procedures
Heart surgery	☐ Yes<br>☐ No<br>☐ Do not know	▢▢ procedures
Hernia surgery (abdomen)	☐ Yes<br>☐ No<br>☐ Do not know	▢▢ procedures
Peritonitis surgery	☐ Yes<br>☐ No<br>☐ Do not know	▢▢ procedures
Removal of kidney stones	☐ Yes<br>☐ No<br>☐ Do not know	▢▢ procedures
Removal of kidney (nephrectomy)	☐ Yes<br>☐ No<br>☐ Do not know	▢▢ procedures
Reduction (partial) of kidney size	☐ Yes<br>☐ No<br>☐ Do not know	▢▢ procedures
Reduction (partial) of liver size	☐ Yes<br>☐ No<br>☐ Do not know	▢▢ procedures
Removal or puncture of non-renal cysts	☐ Yes<br>☐ No<br>☐ Do not know	▢▢ procedures
Removal or puncture of renal cysts	☐ Yes<br>☐ No<br>☐ Do not know	▢▢ procedures
Vascular access (for dialysis)	☐ Yes<br>☐ No<br>☐ Do not know	▢▢ procedures
Other surgery/procedure related to ADPKD or its complications, specify: ____________________	☐ Yes<br>☐ No<br>☐ Do not know	▢▢ procedures

Site ID				Subject ID		

If more room is required than the space provided, please use a separate piece of paper.

19. Over the past 12 months, which of the following diagnostic tests to monitor ADPKD has the subject undergone?

Specify the type and number of diagnostic tests over the past 12 months.

Diagnostic test	Did the patient have a diagnostic test in the past 12 months?	Number of tests over the past 12 months
Abdomen/kidney CT/MRI/PET	☐ Yes<br>☐ No<br>☐ Do not know	__|__ tests
Abdomen/kidney ultrasound	☐ Yes<br>☐ No<br>☐ Do not know	__|__ tests
Blood test	☐ Yes<br>☐ No<br>☐ Do not know	__|__ tests
Brain MRA (angiography)	☐ Yes<br>☐ No<br>☐ Do not know	__|__ tests
Chest X-ray	☐ Yes<br>☐ No<br>☐ Do not know	__|__ tests
Echocardiography	☐ Yes<br>☐ No<br>☐ Do not know	__|__ tests
Urine dipstick test	☐ Yes<br>☐ No<br>☐ Do not know	__|__ tests
Urine lab test	☐ Yes<br>☐ No<br>☐ Do not know	__|__ tests
Other, specify: ________________	☐ Yes<br>☐ No<br>☐ Do not know	__|__ tests
Other, specify: ________________	☐ Yes<br>☐ No<br>☐ Do not know	__|__ tests

If more room is required than the space provided, please use a separate piece of paper.

20. Hospitalisation

Exclude outpatient visits and dialysis visits

a. Over the past 12 months, has the subject been hospitalised directly related to ADPKD or its complications?

☐ Yes
☐ No
☐ Do not know

b. If yes, how many inpatient hospitalisations related to ADPKD and its complications did the subject have in the past 12 months?

|__|__| hospitalisations

c. If yes, how many hospital days did this/these hospitalisation(s) result in?

|__|__| hospital days

21. Outpatient visits

Include all outpatient visits (for example visit with a specialist or follow-up visit).
Exclude dialysis visits.

a. Over the past 12 months, has the subject had any outpatient visits directly related to ADPKD or its complications in the hospital outpatient department?

☐ Yes
☐ No
☐ Do not know

b. If yes, how many outpatient visits did the subject have in the past 12 months?

|__|__| visits

Site ID			Subject ID		

ADPKD-Related Medication Use (Past 12 Months)

22. Hypertension medication

a. In the past 12 months, has the subject been receiving prescriptions provided for hypertension?

- ☐ Yes
- ☐ No
- ☐ Do not know

b. If yes, please provide the details of prescriptions provided for hypertension in the past 12 months.

**Site ID**			**Subject ID**		

Has the subject received the following types of medication in the past 12 months?	If yes, please indicate/write the name of medication(s), and the duration of treatment in the past 12 months (1-52 weeks)
Angiotensin-Converting Enzyme (ACE) inhibitors<br>☐ Yes<br>☐ No<br>☐ Do not know	☐ Enalapril [ ][ ] weeks<br>☐ Other: ____________ [ ][ ] weeks
Angiotensin II receptor antagonists/angiotensin receptor blockers (ARBs)<br>☐ Yes<br>☐ No<br>☐ Do not know	☐ Losartan [ ][ ] weeks<br>☐ Other: ____________ [ ][ ] weeks
Alfa-blockers<br>☐ Yes<br>☐ No<br>☐ Do not know	☐ Name: ____________ [ ][ ] weeks<br>☐ Other: ____________ [ ][ ] weeks
Beta-blockers<br>☐ Yes<br>☐ No<br>☐ Do not know	☐ Metoprolol [ ][ ] weeks<br>☐ Other: ____________ [ ][ ] weeks
Calcium channel blockers/calcium antagonists<br>☐ Yes<br>☐ No<br>☐ Do not know	☐ Amlodipine [ ][ ] weeks

	☐ Felodipine<br>___|___ weeks<br><br>☐ Other: ____________<br>___|___ weeks
Loop diuretics<br><br>☐ Yes<br>☐ No<br>☐ Do not know	☐ Name: ____________<br>___|___ weeks<br><br>☐ Other: ____________<br>___|___ weeks
Thiazides diuretics<br><br>☐ Yes<br>☐ No<br>☐ Do not know	☐ Name: ____________<br>___|___ weeks<br><br>☐ Other: ____________<br>___|___ weeks
Other, not listed above<br><br>☐ Yes<br>☐ No<br>☐ Do not know	☐ Name: ____________<br>___|___ weeks<br><br>☐ Other: ____________<br>___|___ weeks

If more room is required than the space provided, please use a separate piece of paper.

23. Renal pain medication

a. In the past 12 months, has the subject been receiving prescriptions provided for renal pain to manage ADPKD?

☐ Yes
☐ No
☐ Do not know

b. If yes, please provide the details of prescriptions provided for renal pain in the past 12 months.

Has the subject received the following types of medication in the past 12 months?	If yes, please indicate/write the name of medication(s), and the duration of treatment in the past 12 months (1-52 weeks)
Aspirin and nonsteroidal anti-inflammatory drugs, NSAIDS<br><br>☐ Yes<br>☐ No<br>☐ Do not know	☐ Aspirin/acetylsalicylic acid (ASA) ___ ___ weeks<br><br>☐ Ibuprofen/naproxen/COX-2 or other NSAID ___ ___ weeks
Mild analgesics<br><br>☐ Yes<br>☐ No<br>☐ Do not know	☐ Paracetamol ___ ___ weeks<br><br>☐ Other: ____________ ___ ___ weeks
Opioids<br><br>☐ Yes<br>☐ No<br>☐ Do not know	☐ Buprenorphine ___ ___ weeks<br><br>☐ Codeine ___ ___ weeks<br><br>☐ Fentanyl ___ ___ weeks<br><br>☐ Morphine ___ ___ weeks<br><br>☐ Oxycodone ___ ___ weeks<br><br>☐ Tramadol ___ ___ weeks<br><br>☐ Other: ____________ ___ ___ weeks

Other, not listed above<br><br>☐ Yes<br>☐ No<br>☐ Do not know	☐ Name: ____________<br><br>__|__ weeks<br><br>☐ Other: ____________<br><br>__|__ weeks

If more room is required than the space provided, please use a separate piece of paper.

24. Immunosuppressive medication

a. In the past 12 months, has the subject been receiving immunosuppressive medication due to kidney transplant?

☐ Yes
☐ No
☐ Do not know

b. If yes, please provide the details of immunosuppressive medication use in the past 12 months.

Has the subject received the following types of medication in the past 12 months?	If yes, please indicate/write the name of medication(s), and the duration of treatment in the past 12 months (1-52 weeks)
T-cell suppressive agents (CNIs or mTor-1 inhibitors)<br>☐ Yes<br>☐ No<br>☐ Do not know	☐ Ciclosporin (CsA) ___ ___ weeks<br>☐ Sirolimus (SiR) ___ ___ weeks<br>☐ Tacrolimus (TAC) ___ ___ weeks<br>☐ Other: ____________ ___ ___ weeks
Cytotoxic agents (Anti-metabolites)<br>☐ Yes<br>☐ No<br>☐ Do not know	☐ Azathioprine (AZA) ___ ___ weeks<br>☐ Cyclophosphamide ___ ___ weeks<br>☐ Mycophenolate mofetil (MMF) ___ ___ weeks<br>☐ Other: ____________ ___ ___ weeks
Corticosteroids (cortisone)<br>☐ Yes<br>☐ No<br>☐ Do not know	☐ Prednisolone ___ ___ weeks<br>☐ Other: ____________ ___ ___ weeks
Other immunosuppressive	

Has the subject received the following types of medication in the past 12 months?	If yes, please indicate/write the name of medication(s), and the duration of treatment in the past 12 months (1-52 weeks)
medication, not listed above<br><br>☐ Yes<br>☐ No<br>☐ Do not know	☐ Anti-t-lymphocyte globulin, ATG<br>__ __ weeks<br><br>☐ Basiliximab<br>__ __ weeks<br><br>☐ Belatacept<br>__ __ weeks<br><br>☐ Rituximab<br>__ __ weeks<br><br>☐ Other: ____________<br>__ __ weeks

If more room is required than the space provided, please use a separate piece of paper.

25. Other ADPKD-related medications

a. In the past 12 months, has the subject been receiving medications for other ADPKD-related conditions?

☐ Yes
☐ No
☐ Do not know

b. If yes, please provide the details of prescriptions provided for other ADPKD conditions in the past 12 months.

Has the subject received the following types of medication in the past 12 months?	If yes, please indicate/write the name of medication(s), and the duration of treatment in the past 12 months (1-52 weeks)
Phosphate binders<br>☐ Yes<br>☐ No<br>☐ Do not know	☐ Sevelamer ⎣__⎦⎣__⎦ weeks<br>☐ Lanthanum carbonate ⎣__⎦⎣__⎦ weeks<br>☐ Calcium acetate/ magnesium carbonate ⎣__⎦⎣__⎦ weeks<br>☐ Colestilan ⎣__⎦⎣__⎦ weeks<br>☐ Other: ____________ ⎣__⎦⎣__⎦ weeks

Type of prescription	Has the subject received this type of prescription in the past 12 months?	Duration of treatment during the past 12 months (1-52 weeks)
Antibiotics	☐ Yes<br>☐ No<br>☐ Do not know	__ __ weeks
Antivirals	☐ Yes<br>☐ No<br>☐ Do not know	__ __ weeks
Bisphosphonates	☐ Yes<br>☐ No<br>☐ Do not know	__ __ weeks
Erythropoiesis-Stimulating Agents (ESAs)	☐ Yes<br>☐ No<br>☐ Do not know	__ __ weeks
Iron, oral	☐ Yes<br>☐ No<br>☐ Do not know	__ __ weeks
Iron, parenteral (IV)	☐ Yes<br>☐ No<br>☐ Do not know	__ __ weeks
Vitamin D analogs	☐ Yes<br>☐ No<br>☐ Do not know	__ __ weeks
Other, specify: __________________	☐ Yes<br>☐ No<br>☐ Do not know	__ __ weeks

If more room is required than the space provided, please use a separate piece of paper.

Dialysis-Related Resource Use (Past 12 Months)

26. Regular dialysis

Regular dialysis refers to dialysis due to chronic kidney disease (i.e. not temporary dialysis).
Self-care-dialysis refers to when the patient manages dialysis at home/work.

a. Over the past 12 months, has the subject received regular dialysis?

☐ Yes
☐ No
☐ Do not know

b. If yes, please indicate, during the past 12 months, the initial type of dialysis, regimen, and duration.

Type of dialysis	Where did the subject receive dialysis in the past 12 months?	Frequency of dialysis per week (1-7 times/week) or type of PD	Duration of being on dialysis during the past 12 months (1-52 weeks)
Haemodialysis (HD)<br>☐ Yes<br>☐ No<br>☐ Do not know	☐ Home/Self-care<br>☐ Home/Assisted<br>☐ At clinic/hospital	per week	weeks
Peritoneal dialysis (PD)<br>☐ Yes<br>☐ No<br>☐ Do not know	☐ Home/Self-care<br>☐ Home/Assisted<br>☐ At clinic/hospital	☐ Continuous ambulatory peritoneal dialysis (CAPD)<br>☐ Automated peritoneal dialysis (APD)<br>☐ Do not know	weeks
If the patient changed type of dialysis or frequency during the past 12 months, please indicate the new type/frequency/duration below:			
Type of dialysis	Where did the subject receive dialysis in the past 12 months?	Frequency of dialysis per week (1-7 times/week) or type of PD	Duration of being on dialysis during the past 12 months (1-52 weeks)
Haemodialysis (HD)<br>☐ Yes<br>☐ No<br>☐ Do not know	☐ Home/Self-care<br>☐ Home/Assisted<br>☐ At clinic/hospital	per week	weeks
Peritoneal dialysis (PD)<br>☐ Yes<br>☐ No<br>☐ Do not know	☐ Home/Self-care<br>☐ Home/Assisted<br>☐ At clinic/hospital	☐ Continuous ambulatory peritoneal dialysis (CAPD)<br>☐ Automated peritoneal dialysis (APD)<br>☐ Do not know	weeks

If more room is required than the space provided, please use a separate piece of paper.

Additional Questions in Part II and III

The next two parts (Part II and Part III) collect additional information on subjects enrolled into the Dialysis and Transplant stratum, respectively.

27. Is the subject enrolled into the:

a. … *Dialysis stratum* (on dialysis initiated at least 6 months ago; with/without transplanted kidney)?

- ☐ Yes (continue with *Q28*)
- ☐ No

b. …*Transplant stratum* (working transplanted kidney since at least 6 months ago; not on dialysis)?

- ☐ Yes (skip *Part II* and continue with *Q33*)
- ☐ No

c. … *CDK stage 4–5* or *CDK stage 1–3* stratum (not currently on dialysis/no previous transplant)?

- ☐ Yes (skip *Parts II and III* and continue to the last page, *Q40*)
- ☐ No

Site ID				Subject ID		

Part II
Period around
Initiation of Dialysis

Strata: *Dialysis*

Dialysis-Related Resource Use (Period around Initiation of Dialysis)

This following section refers to resource use around the time of initiation of regular dialysis (6 months before until 6 months after dialysis initiation).
If the patient has been on and off regular dialysis, refer to the most recent time the patient initiated dialysis.

28. Days of interest around start of dialysis

a. What was the date of dialysis initiation?

Dialysis date

__ __ / __ __ / __ __ __ __
D D M M Y Y Y Y

b. Subtract 6 months from the dialysis date above

Used as start date for data collection around dialysis start

Date 6 months <u>before</u> dialysis
(Start date)

__ __ / __ __ / __ __ __ __
D D M M Y Y Y Y

c. Add 6 months to the dialysis date above

Used as end date for data collection around dialysis start

Date 6 months <u>after</u> dialysis
(End date)

__ __ / __ __ / __ __ __ __
D D M M Y Y Y Y

29. Resource use around dialysis start

In the table that follows, please state the subject's hospitalisations and outpatient visits in the period around dialysis start. Please note that the first part of the table refers to the period before dialysis start whereas the second part of the table refers to the period after dialysis start.

Site ID	Subject ID
☐☐☐	☐☐☐

	Has the subject had the resource use type described below?	If yes, how many hospitalisations/ outpatient visits?	If any hospitalisations, how many hospital days did this/these hospitalisation/s result in?
Before dialysis start, i.e. period 6 months before dialysis start (Start date–Dialysis Date)			
Hospitalisations<br><br>*Exclude outpatient visits*	☐ Yes<br>☐ No<br>☐ Do not know	☐☐ hospitalisations	☐☐ days
Outpatient visits<br><br>*Exclude hospitalisations*	☐ Yes<br>☐ No<br>☐ Do not know	☐☐ visits	
After dialysis start, i.e. the period 6 months after dialysis start (Dialysis Date–End Date)			
Hospitalisations<br><br>*Exclude outpatient visits*<br>*Also exclude dialysis visits*	☐ Yes<br>☐ No<br>☐ Do not know	☐☐ hospitalisations	☐☐ days
Outpatient visits<br><br>*Exclude hospitalisations*<br>*Also exclude dialysis visits*	☐ Yes<br>☐ No<br>☐ Do not know	☐☐ visits	

30. Indicate the initial type of dialysis and regimen during the 6 months after dialysis start.

Type of dialysis	Where did the subject receive dialysis?	Frequency of dialysis per week (1-7 times/week) or type of PD	Duration of being on dialysis (1-26 weeks)
Haemodialysis (HD)<br>☐ Yes<br>☐ No<br>☐ Do not know	☐ Home/Self-care<br>☐ Home/Assisted<br>☐ At clinic/hospital	⎿⎿ per week	⎿⎿⎿ weeks
Peritoneal dialysis (PD)<br>☐ Yes<br>☐ No<br>☐ Do not know	☐ Home/Self-care<br>☐ Home/Assisted<br>☐ At clinic/hospital	☐ Continuous ambulatory peritoneal dialysis (CAPD)<br>☐ Automated peritoneal dialysis (APD)<br>☐ Do not know	⎿⎿⎿ weeks
If the patient changed the type of dialysis or frequency in the 6-month period after dialysis start, please indicate the new type/frequency/duration below:			
Type of dialysis	Where did the subject receive dialysis?	Frequency of dialysis per week (1-7 times/week) or type of PD	Duration of being on dialysis (1-26 weeks)
Haemodialysis (HD)<br>☐ Yes<br>☐ No<br>☐ Do not know	☐ Home/Self-care<br>☐ Home/Assisted<br>☐ At clinic/hospital	⎿⎿ per week	⎿⎿⎿ weeks
Peritoneal dialysis (PD)<br>☐ Yes<br>☐ No<br>☐ Do not know	☐ Home/Self-care<br>☐ Home/Assisted<br>☐ At clinic/hospital	☐ Continuous ambulatory peritoneal dialysis (CAPD)<br>☐ Automated peritoneal dialysis (APD)<br>☐ Do not know	⎿⎿⎿ weeks

If more room is required than the space provided, please use a separate piece of paper.

31. Medical surgeries/treatment procedures in the 6 months before dialysis start

a. In the 6 months before dialysis start, did the subject have any medical surgeries/treatment procedures?

☐ Yes
☐ No
☐ Do not know

b. If yes, please provide details below.

Surgery/procedure	Did the patient have the procedure in the 6 months before dialysis start?	Number of procedures in the 6-month period
Acute blood transfusion	☐ Yes<br>☐ No<br>☐ Do not know	☐☐ procedures
Brain aneurysm procedure	☐ Yes<br>☐ No<br>☐ Do not know	☐☐ procedures
Heart surgery	☐ Yes<br>☐ No<br>☐ Do not know	☐☐ procedures
Hernia surgery (abdomen)	☐ Yes<br>☐ No<br>☐ Do not know	☐☐ procedures
Peritonitis surgery	☐ Yes<br>☐ No<br>☐ Do not know	☐☐ procedures
Removal of kidney stones	☐ Yes<br>☐ No<br>☐ Do not know	☐☐ procedures
Removal of kidney (nephrectomy)	☐ Yes<br>☐ No<br>☐ Do not know	☐☐ procedures
Reduction (partial) of kidney size	☐ Yes<br>☐ No<br>☐ Do not know	☐☐ procedures
Reduction (partial) of liver size	☐ Yes<br>☐ No<br>☐ Do not know	☐☐ procedures
Removal or puncture of non-renal cysts	☐ Yes<br>☐ No<br>☐ Do not know	☐☐ procedures
Removal or puncture of renal cysts	☐ Yes<br>☐ No<br>☐ Do not know	☐☐ procedures
Vascular access (for dialysis)	☐ Yes<br>☐ No<br>☐ Do not know	☐☐ procedures
Other surgery/procedure related to ADPKD or its complications, specify:<br>________________________	☐ Yes<br>☐ No<br>☐ Do not know	☐☐ procedures

Site ID				Subject ID		

If more room is required than the space provided, please use a separate piece of paper.

32. Medical surgeries/treatment procedures in the 6 months after dialysis start

a. In the 6 months after dialysis start, did the subject have any medical surgeries/treatment procedures?

- ☐ Yes
- ☐ No
- ☐ Do not know

b. If yes, please provide details below.

Surgery/procedure	Did the patient have the procedure in the 6 months after dialysis start?	Number of procedures in the 6-month period
Acute blood transfusion	☐ Yes<br>☐ No<br>☐ Do not know	☐☐ procedures
Brain aneurysm procedure	☐ Yes<br>☐ No<br>☐ Do not know	☐☐ procedures
Heart surgery	☐ Yes<br>☐ No<br>☐ Do not know	☐☐ procedures
Hernia surgery (abdomen)	☐ Yes<br>☐ No<br>☐ Do not know	☐☐ procedures
Peritonitis surgery	☐ Yes<br>☐ No<br>☐ Do not know	☐☐ procedures
Removal of kidney stones	☐ Yes<br>☐ No<br>☐ Do not know	☐☐ procedures
Removal of kidney (nephrectomy)	☐ Yes<br>☐ No<br>☐ Do not know	☐☐ procedures
Reduction (partial) of kidney size	☐ Yes<br>☐ No<br>☐ Do not know	☐☐ procedures
Reduction (partial) of liver size	☐ Yes<br>☐ No<br>☐ Do not know	☐☐ procedures
Removal or puncture of non-renal cysts	☐ Yes<br>☐ No<br>☐ Do not know	☐☐ procedures
Removal or puncture of renal cysts	☐ Yes<br>☐ No<br>☐ Do not know	☐☐ procedures
Vascular access (for dialysis)	☐ Yes<br>☐ No<br>☐ Do not know	☐☐ procedures
Other surgery/procedure related to ADPKD or its complications,<br>specify:<br>______________________	☐ Yes<br>☐ No<br>☐ Do not know	☐☐ procedures

Site ID				Subject ID		

If more room is required than the space provided, please use a separate piece of paper.

If you have filled out this section for a subject enrolled into the Dialysis strata, please skip Part III and continue to the last page (Q40).

Site ID				Subject ID		

Part III: Period around Transplant Operation

Strata: *Transplant*

Kidney Transplant-Related Resource Use (Period around the Transplant Operation)

This section refers to resource use around the time of kidney transplant.
If the patient has been re-transplanted, refer to the most recent kidney transplant.

33. Days of interest around transplant

a. What was the date of transplant?

Transplant date

__ __ / __ __ / __ __ __ __
D D M M Y Y Y Y

b. Subtract 6 months from the transplant date above

Used as start date for data collection around transplant

Date 6 months <u>before</u> transplant
(Start date)

__ __ / __ __ / __ __ __ __
D D M M Y Y Y Y

c. Add 6 months to the transplant date above

Used as end date for data collection around transplant

Date 6 months <u>after</u> transplant
(End date)

__ __ / __ __ / __ __ __ __
D D M M Y Y Y Y

34. Resource use around transplantation

In the below table please state the subject's hospitalisations and outpatient visits in the period around transplantation. Please note that the first part of the table refers to the period <u>before</u> transplantation whereas the second part of the table refers to the period <u>after</u> the subject had his/her transplant.

	Has the subject had the resource use type described below?	If yes, how many hospitalisations/ outpatient visits?	If any hospitalisations, how many hospital days did this/these hospitalisation/s result in?
Before transplantation, i.e. period 6 months before transplantation (Start date–Transplant Date)			
Hospitalisations<br><br>*Exclude outpatient visits*<br>*Also exclude dialysis visits*	☐ Yes<br>☐ No<br>☐ Do not know	|__|__| hospitalisations	|__|__| days
Outpatient visits<br><br>*Exclude hospitalisations*<br>*Also exclude dialysis visits*	☐ Yes<br>☐ No<br>☐ Do not know	|__|__| visits	
After transplantation, i.e. the period 6 months after transplantation (Transplant Date–End Date)			
Hospitalisations<br><br>*Exclude outpatient visits*<br>*Also exclude dialysis visits*	☐ Yes<br>☐ No<br>☐ Do not know	|__|__| hospitalisations	|__|__| days
Outpatient visits<br><br>*Exclude hospitalisations*<br>*Also exclude dialysis visits*	☐ Yes<br>☐ No<br>☐ Do not know	|__|__| visits	

35. Medical surgeries/treatment procedures in the 6 months before transplantation

a. In the 6 months before transplantation, did the subject have any medical surgeries/treatment procedures?

☐ Yes
☐ No
☐ Do not know

b. If yes, please provide details below.

Surgery/procedure	Did the patient have the procedure in the 6 months before transplantation?	Number of procedures in the 6-month period
Acute blood transfusion	☐ Yes ☐ No ☐ Do not know	☐☐ procedures
Brain aneurysm procedure	☐ Yes ☐ No ☐ Do not know	☐☐ procedures
Heart surgery	☐ Yes ☐ No ☐ Do not know	☐☐ procedures
Hernia surgery (abdomen)	☐ Yes ☐ No ☐ Do not know	☐☐ procedures
Peritonitis surgery	☐ Yes ☐ No ☐ Do not know	☐☐ procedures
Removal of kidney stones	☐ Yes ☐ No ☐ Do not know	☐☐ procedures
Removal of kidney (nephrectomy)	☐ Yes ☐ No ☐ Do not know	☐☐ procedures
Reduction (partial) of kidney size	☐ Yes ☐ No ☐ Do not know	☐☐ procedures
Reduction (partial) of liver size	☐ Yes ☐ No ☐ Do not know	☐☐ procedures
Removal or puncture of non-renal cysts	☐ Yes ☐ No ☐ Do not know	☐☐ procedures
Removal or puncture of renal cysts	☐ Yes ☐ No ☐ Do not know	☐☐ procedures
Vascular access (for dialysis)	☐ Yes ☐ No ☐ Do not know	☐☐ procedures
Other surgery/procedure related to ADPKD or its complications, specify: ______________________	☐ Yes ☐ No ☐ Do not know	☐☐ procedures

If more room is required than the space provided, please use a separate piece of paper.

36. Dialysis before transplantation

a. In the 6 months before transplantation, did the subject receive regular dialysis?

- ☐ Yes
- ☐ No
- ☐ Do not know

b. If yes, please indicate the initial type of dialysis and regimen during the 6 months before transplantation

Type of dialysis	Where did the subject receive dialysis?	Frequency of dialysis per week (1-7 times/week) or type of PD	Duration of being on dialysis (1-26 weeks)
Haemodialysis (HD)<br><br>☐ Yes<br>☐ No<br>☐ Do not know	☐ Home/Self-care<br>☐ Home/Assisted<br>☐ At clinic/hospital	___ per week	___ ___ weeks
Peritoneal dialysis (PD)<br><br>☐ Yes<br>☐ No<br>☐ Do not know	☐ Home/Self-care<br>☐ Home/Assisted<br>☐ At clinic/hospital	☐ Continuous ambulatory peritoneal dialysis (CAPD)<br>☐ Automated peritoneal dialysis (APD)<br>☐ Do not know	___ ___ weeks

If more room is required than the space provided, please use a separate piece of paper.

37. Medical surgeries/treatment procedures in the 6 months after transplantation

a. In the 6 months after transplantation, did the subject have any medical surgeries/treatment procedures?

- ☐ Yes
- ☐ No
- ☐ Do not know

b. If yes, please provide details below.

Surgery/procedure	Did the patient have the procedure in the 6 months after transplantation?	Number of procedures in the 6-month period
Acute blood transfusion	☐ Yes<br>☐ No<br>☐ Do not know	procedures
Brain aneurysm procedure	☐ Yes<br>☐ No<br>☐ Do not know	procedures
Heart surgery	☐ Yes<br>☐ No<br>☐ Do not know	procedures
Hernia surgery (abdomen)	☐ Yes<br>☐ No<br>☐ Do not know	procedures
Peritonitis surgery	☐ Yes<br>☐ No<br>☐ Do not know	procedures
Removal of kidney stones	☐ Yes<br>☐ No<br>☐ Do not know	procedures
Removal of kidney (nephrectomy)	☐ Yes<br>☐ No<br>☐ Do not know	procedures
Reduction (partial) of kidney size	☐ Yes<br>☐ No<br>☐ Do not know	procedures
Reduction (partial) of liver size	☐ Yes<br>☐ No<br>☐ Do not know	procedures
Removal or puncture of non-renal cysts	☐ Yes<br>☐ No<br>☐ Do not know	procedures
Removal or puncture of renal cysts	☐ Yes<br>☐ No<br>☐ Do not know	procedures
Vascular access (for dialysis)	☐ Yes<br>☐ No<br>☐ Do not know	procedures
Other surgery/procedure related to ADPKD or its complications, specify: ______________________	☐ Yes<br>☐ No<br>☐ Do not know	procedures

If more room is required than the space provided, please use a separate piece of paper.

38. Dialysis after transplantation

a. In the 6 months after transplantation, did the subject receive regular dialysis?

☐ Yes
☐ No
☐ Do not know

b. If yes, please indicate the initial type of dialysis and regimen during the 6 months after transplantation

Type of dialysis	Where did the subject receive dialysis?	Frequency of dialysis per week (1-7 times/week) or type of PD	Duration of being on dialysis (1-26 weeks)
Haemodialysis (HD)<br>☐ Yes<br>☐ No<br>☐ Do not know	☐ Home/Self-care<br>☐ Home/Assisted<br>☐ At clinic/hospital	__ per week	__ __ weeks
Peritoneal dialysis (PD)<br>☐ Yes<br>☐ No<br>☐ Do not know	☐ Home/Self-care<br>☐ Home/Assisted<br>☐ At clinic/hospital	☐ Continuous ambulatory peritoneal dialysis (CAPD)<br>☐ Automated peritoneal dialysis (APD)<br>☐ Do not know	__ __ weeks

If more room is required than the space provided, please use a separate piece of paper.

39. Immunosuppressive medication

a. In the 6 months after transplant, did the subject receive immunosuppressive medication?

☐ Yes
☐ No
☐ Do not know

b. If yes, please provide the details of the immunosuppressive medication provided in the 6 months after transplant.

Has the subject received the following types of medication during the 6-month period?	If yes, please indicate/write the name of medication(s), and the duration of treatment during the 6-month period (1-26 weeks)
T-cell suppressive agents (CNIs or mTor-1 inhibitors)<br>☐ Yes<br>☐ No<br>☐ Do not know	☐ Ciclosporin (CsA) __|__ weeks<br>☐ Sirolimus (SiR) __|__ weeks<br>☐ Tacrolimus (TAC) __|__ weeks<br>☐ Other: ____________ __|__ weeks
Cytotoxic agents (Anti-metabolites)<br>☐ Yes<br>☐ No<br>☐ Do not know	☐ Azathioprine (AZA) __|__ weeks<br>☐ Cyclophosphamide __|__ weeks<br>☐ Mycophenolate mofetil (MMF) __|__ weeks<br>☐ Other: ____________ __|__ weeks
Corticosteroids (cortisone)<br>☐ Yes<br>☐ No<br>☐ Do not know	☐ Prednisolone __|__ weeks<br>☐ Other: ____________ __|__ weeks

Site ID				Subject ID		

Has the subject received the following types of medication during the 6-month period?	If yes, please indicate/write the name of medication(s), and the duration of treatment during the 6-month period (1-26 weeks)
Other immunosuppressive medication, not listed above<br><br>☐ Yes<br>☐ No<br>☐ Do not know	☐ Anti-t-lymphocyte globulin, ATG<br>__ __ weeks<br><br>☐ Basiliximab<br>__ __ weeks<br><br>☐ Belatacept<br>__ __ weeks<br><br>☐ Rituximab<br>__ __ weeks<br><br>☐ Other: ____________<br>__ __ weeks

If more room is required than the space provided, please use a separate piece of paper

Site ID				Subject ID		

Signature: Investigator or Nominated Research Staff

40. I have reviewed all pages of this CRF and certify that they are accurate and complete:

Name: ______________________________

Signature: ______________________________

Date: __ __ / __ __ / __ __ __ __
D D M M Y Y Y Y
