# Supplementary material for: Real-world costs of autosomal dominant polycystic kidney disease in the Nordics
Source: BMC Health Serv Res. 2017 Aug 15;17:560. doi: 10.1186/s12913-017-2513-8 (PMC5556351; doi:10.1186/s12913-017-2513-8)
Supplement: Supplementary file 2 — QR ADPKD Additional Questions 20140205 Eng.pdf – Self-administered questionnaire – Questionnaire administered to patients. (PDF 16 kb) [file 12913_2017_2513_MOESM2_ESM.pdf]

Site ID			Subject Number		

Questionnaire

Instructions and clarifications appear throughout this form in italic

Introduction

In this questionnaire, we ask you to provide information related to your cystic kidney disease. This information is crucial to the understanding of the disease. Primarily we are interested in complementing the data from your specialist clinic with information on other cystic kidney disease-related care and also with information on travel costs and help you may receive around your home.

Questionnaire

1. What type of residence do you live in?

   - ☐ House/apartment
   - ☐ Nursing home
   - ☐ Other, please specify: ________________

2. Help around the home from family and friends

   a. During the past four weeks, have you due to your cystic kidney disease received help around the home from family and/or friends?

   - ☐ Yes
   - ☐ No

   b. <u>If yes</u>, on average during the past four weeks, how many hours per week did you get help?

   |__|__| hours per week

3. Home care service

   a. During the past four weeks, have you due to your cystic kidney disease received home care service?

   - ☐ Yes
   - ☐ No

   b. <u>If yes</u>, on average during the past four weeks, how many hours per week did you get help?

   |__|__| hours per week

4. Doctor/nurse home visit

   a. During the past four weeks have you, due to your cystic kidney disease, received help in your home from a doctor, nurse or other health care professional?

   - ☐ Yes
   - ☐ No

   b. <u>If yes</u>, how many times during the past four weeks did you receive a home visit?

   |__|__| times

5. During the past four weeks, due to your cystic kidney disease, how many visits have you made to the following...

   For each question below, please write “0” if you have not had the visit.

a. ... nephrology clinic other than your kidney specialist clinic from which you received this questionnaire?

|__|__| visits

b. … physician/nurse office (primary care) outside of your nephrology clinic (kidney specialist)?

|__|__| visits

c. … physiotherapist?

|__|__| visits

d. … psychologist/psychiatrist?

|__|__| visits

e. ...alternative medicine clinic (for example, massage, acupuncture, chiropractic therapy, osteopathy, etc.)?

|__|__| visits

f. ...other specialist care (not listed above)?

|__|__| visits

6. During the past four weeks, due to cystic kidney disease, to what amount have you bought over-the-counter drugs (i.e. drugs not requiring a prescription)?

|__|__|__|__| DKK/EURO/NOK/SEK

7. Dialysis

a. Do you regularly travel to a dialysis unit for dialysis?

- ☐ Yes
- ☐ No

b. <u>If yes</u>, what type of transport do you usually use to travel to your dialysis unit?

- ☐ Public transportation
- ☐ Car
- ☐ Taxi/Community transport
- ☐ Walking
- ☐ Other, please specify: ________________

c. <u>If yes</u>, how long does it take for you to travel to the dialysis unit?

- ☐ less than 30 min
- ☐ 30 min to 1 hour
- ☐ 1 to 2 hours
- ☐ longer than 2 hours
